# Profiling morphologic MRI features of motor neuron disease caused by *TARDBP* mutations

**DOI:** 10.3389/fneur.2022.931006

**Published:** 2022-07-15

**Authors:** Edoardo Gioele Spinelli, Alma Ghirelli, Nilo Riva, Elisa Canu, Veronica Castelnovo, Teuta Domi, Laura Pozzi, Paola Carrera, Vincenzo Silani, Adriano Chiò, Massimo Filippi, Federica Agosta

**Affiliations:** ^1^Neuroimaging Research Unit, Division of Neuroscience, IRCCS San Raffaele Scientific Institute, Milan, Italy; ^2^Neurology Unit, IRCCS San Raffaele Scientific Institute, Milan, Italy; ^3^Vita-Salute San Raffaele University, Milan, Italy; ^4^Neurorehabilitation Unit, IRCCS San Raffaele Scientific Institute, Milan, Italy; ^5^Experimental Neuropathology Unit, Division of Neuroscience, IRCCS San Raffaele Scientific Institute, Milan, Italy; ^6^Laboratory of Clinical Molecular Biology, Unit of Genomics for Human Disease Diagnosis, Division of Genetics and Cell Biology, IRCCS Ospedale San Raffaele, Milan, Italy; ^7^Department of Neurology and Laboratory of Neuroscience, IRCCS Istituto Auxologico Italiano, Milan, Italy; ^8^“Dino Ferrari” Center, Department of Pathophysiology and Transplantation, Università degli Studi di Milano, Milan, Italy; ^9^Rita Levi Montalcini “Department of Neuroscience, ” ALS Center, University of Torino, Turin, Italy; ^10^Neurophysiology Service, IRCCS San Raffaele Scientific Institute, Milan, Italy

**Keywords:** motor neuron disease (MND), amyotrophic lateral sclerosis (ALS), *transactive response (TAR) DNA binding protein 43 (TARDBP)*, magnetic resonance imaging (MRI), voxel-based morphometry (VBM)

## Abstract

**Objective:**

Mutations in the *TARDBP* gene are a rare cause of genetic motor neuron disease (MND). Morphologic MRI characteristics of MND patients carrying this mutation have been poorly described. Our objective was to investigate distinctive clinical and MRI features of a relatively large sample of MND patients carrying *TARDBP* mutations.

**Methods:**

Eleven MND patients carrying a *TARDBP* mutation were enrolled. Eleven patients with sporadic MND (sMND) and no genetic mutations were also selected and individually matched by age, sex, clinical presentation and disease severity, along with 22 healthy controls. Patients underwent clinical and cognitive evaluations, as well as 3D T1-weighted and diffusion tensor (DT) MRI on a 3 Tesla scanner. Gray matter (GM) atrophy was first investigated at a whole-brain level using voxel-based morphometry (VBM). GM volumes and DT MRI metrics of the main white matter (WM) tracts were also obtained. Clinical, cognitive and MRI features were compared between groups.

**Results:**

MND with *TARDBP* mutations was associated with all possible clinical phenotypes, including isolated upper/lower motor neuron involvement, with no predilection for bulbar or limb involvement at presentation. Greater impairment at naming tasks was found in TARDBP mutation carriers compared with sMND. VBM analysis showed significant atrophy of the right lateral parietal cortex in *TARDBP* patients, compared with controls. A distinctive reduction of GM volumes was found in the left precuneus and right angular gyrus of *TARDBP* patients compared to controls. WM microstructural damage of the corticospinal tract (CST) and inferior longitudinal fasciculi (ILF) was found in both sMND and *TARDBP* patients, compared with controls, although decreased fractional anisotropy of the right CST and increased axial diffusivity of the left ILF (*p* = 0.017) was detected only in *TARDBP* mutation carriers.

**Conclusions:**

*TARDBP* patients showed a distinctive parietal pattern of cortical atrophy and greater damage of motor and extra-motor WM tracts compared with controls, which sMND patients matched for disease severity and clinical presentation were lacking. Our findings suggest that TDP-43 pathology due to *TARDBP* mutations may cause deeper morphologic alterations in both GM and WM.

## Introduction

Motor neuron disease (MND) refers to a group of adult-onset neurodegenerative conditions leading to the degeneration of upper and/or lower motor neurons. MND clinically manifests as a progressive loss of motor function, which ultimately leads to death due to the involvement of respiratory muscles (1). Amyotrophic lateral sclerosis (ALS) is the classic form of MND and is characterized by a combination of signs and symptoms of upper and lower motor neuron involvement. However, pure lower motor neuron [i.e., primary muscular atrophy (PMA)] or upper motor neuron [i.e., primary lateral sclerosis (PLS)] involvement can also be appreciated in a minority of cases (2).

One out of ten ALS patients has a familial form, the remaining cases being sporadic (3, 4). Mutations of more than 20 genes have been found to cause MND (5), the most common being *chromosome 9 open reading frame 72 (C9Orf72)* (33% of familial ALS) and *superoxidase dismutase 1 (SOD1)* (14% of familial ALS) (6). The third genetic cause of ALS in terms of frequency is the mutation in the *transactive response (TAR) DNA binding protein 43 (TARDBP)* gene (4.2% of familial cases) (6–9), which encodes the TDP-43 protein. In normal conditions, TDP-43 is ubiquitously expressed at the nuclear level, where it has a role in regulating transcription, messenger RNA (mRNA) spicing and transport, as well as in scaffolding nuclear bodies during interaction with the survival motor neuron protein (10, 11). In the central nervous system of MND patients, an abnormal accumulation of toxic aggregates of hyperphosphorylated TDP-43 is typically observed in the cytoplasm of motor neurons (12). *TARDBP* mutations have been described in a few cases of ALS, frontotemporal dementia (FTD), or combined presentations thereof (13–22), and are thought to facilitate or accelerate such neuropathological alterations. Consistent with this hypothesis, *TARDBP* mutations have been associated with increased TDP-43 intracellular aggregation, aberrant cytoplasmic localization, altered protein stability, resistance to protease action or modified interactions with other proteins (11, 13, 23–26). Furthermore, the level of TDP-43 that accumulates in granules, as well as the size or number of granules appear to be bigger in some forms of genetic ALS compared to sporadic cases (27, 28). Different mutations associated with familial ALS have been demonstrated to increase half-life and improve stability of the TDP-43 protein product compared to the wild-type form (29). This could be a potential mechanism for the accelerated disease onset of familial ALS (30, 31).

On magnetic resonance imaging (MRI), no typical pattern of atrophy has emerged in *TARDBP* patients, probably due to the limited number of cases described. Patterns of atrophy were mostly consistent with the individual clinical presentation, since cases of behavioral and linguistic variants of FTD associated with *TARDBP* mutations have shown variable combinations of temporal and/or frontal lobe involvement (14, 16, 18, 21), whereas cases of ALS associated with *TARDBP* mutation reported in literature presented either a normal MRI (15, 17, 32, 33) or mild bilateral temporal atrophy (33). One reported case of behavioral variant of FTD presented also with mesencephalic and caudate nuclei atrophy (19) in the presence of supranuclear gaze palsy and chorea. This far, no study has analyzed *TARDBP* mutated patients with advanced MRI techniques. The aim of this work was to investigate distinctive clinical and MRI features of a relatively large sample of 11 MND patients carrying *TARDBP* mutations.

## Materials and methods

### Participants

A total of 379 patients with a confirmed diagnosis of MND were referred to IRCCS San Raffaele Scientific Institute in Milan between October 2007 and November 2021 to perform an MRI scan on a 3 Tesla scanner. Their diagnoses included ALS (34–36), PMA (34–36) and PLS (34–36). Patients were screened for known pathogenic mutations on the *C9Orf72, GRN, MAPT, FUS, TARDBP, SOD1, TBK1, TREM2, OPTN*, or *VCP* genes. As a result of the screening process, 11 *TARDBP* mutation carriers were identified, all presenting a pure MND phenotype (ALS, *n* = 7; PMA, *n* = 3; PLS, *n* = 1). As a control group, we included 11 MND patients [sporadic MND (sMND); ALS, *n* = 8; PMA, *n* = 3) who proved negative for known pathogenic mutations or variants of unknown significance on the evaluated MND-related genes, and were matched by age, sex, clinical presentation, and disease severity as measured by the ALS Functional Rating Scale Revised (ALSFRS-r) (37), as best as possible on a one-to-one basis. All patients underwent a thorough neurological examination and brain MRI at study entry, as well as a comprehensive, multi-domain clinical, cognitive and behavioral assessment. Twenty-two healthy controls matched for age, sex, and MRI scanner type were recruited among spouses of patients and by word of mouth. Healthy controls were included if the following criteria were satisfied: normal neurological assessment; MMSE score ≥28; no family history of neurodegenerative diseases. All included subjects (i.e., ALS patients and healthy controls) were right-handed, Caucasian, native Italian speakers. Exclusion criteria for all subjects were: significant medical illnesses or substance abuse that could interfere with cognitive functioning; any (other) major systemic, psychiatric, or neurological illnesses; and other causes of focal or diffuse brain damage, including lacunae and extensive cerebrovascular disorders at routine MRI.

Local ethical standards committee on human experimentation approved the study protocol and all participants provided written informed consent.

### Genetic analysis

Blood samples were collected from all patients. The coding sequences and intron/exon boundaries of *TARDBP* gene were amplified by PCR using optimized protocols and analyzed using Sanger sequencing, looking for known pathogenic mutations (38). Furthermore, the presence of GGGGCC hexanucleotide expansion in the first intron of *C9Orf72* was assessed using a repeat-primed polymerase chain reaction (PCR) assay (39). The coding sequences and intron/exon boundaries of *GRN, MAPT, SOD1, FUS, TBK1, TREM2, OPTN*, and *VCP* genes were also evaluated by Sanger sequencing (38).

### Clinical evaluation

Clinical evaluation was performed by experienced neurologists blinded to genetic status and MRI results, recording disease duration and site of disease onset. Disease severity was assessed using the ALSFRS-r (37). The rate of disease progression was defined according to the following formula: (48–ALSFRS-r score)/time from symptom onset. Muscular strength was assessed by manual muscle testing based on the Medical Research Council (MRC) scale.

### Neuropsychological evaluation

Neuropsychological assessment was performed by an experienced neuropsychologist unaware of genetic status and MRI results. The following cognitive functions were evaluated: global cognitive functioning with the MMSE (40); long- and short-term verbal memory with the Rey Auditory Verbal Learning Test (41) and the digit span forward (42), respectively; attentive and executive functions with the digit span backward (43) and the Ravens colored progressive matrices (44); fluency with the phonemic and semantic fluency tests (45) and the relative fluency indices (controlling for individual motor disabilities) (46); visuospatial abilities with the Rey Figure copy (47); language with the Italian battery for the assessment of aphasic disorders (48) and the Token test (49); mood and the presence of behavioral disturbances with the Frontal Behavioral Inventory (50). Although most patients here included were enrolled prior to the revision of Strong criteria (51) and the validation of the Edinburgh Cognitive and Behavioral ALS Screen (ECAS) scale (52), for 11 patients (six mutation carriers and five sMND), sufficient neuropsychological data were available to make a diagnosis of cognitive and/or behavioral impairment according to the revised Strong criteria (51).

### MRI acquisition

All patients and healthy controls underwent brain MRI on a 3.0 T scanner (Philips Medical Systems, Best, the Netherlands) at IRCSS San Raffaele Scientific Institute between 2007 and 2021. The original scanner (for brevity, Scanner 1) was substituted with a different device from the same manufacturer in 2016 (below defined as Scanner 2).

Using Scanner 1, the following brain MRI sequences were obtained: T2-weighted spin echo (SE) [repetition time (TR) = 3,500 ms; echo time (TE) = 85 ms; echo train length = 15; flip angle = 90; 22 contiguous, 5-mm-thick, axial slices; matrix size = 512 × 512; field of view (FOV) = 230 × 184 mm^2^]; fluid-attenuated inversion recovery (TR = 11 s; TE = 120 ms; flip angle = 90; 22 contiguous, 5-mm-thick, axial slices; matrix size = 512 × 512; FOV = 230 mm^2^); and 3D T1-weighted fast field echo (TR = 25 ms, TE = 4.6 ms, flip angle = 30, 220 contiguous axial slices with voxel size = 0.89 × 0.89 × 0.8 mm, matrix size = 256 × 256, FOV = 230 × 182 mm^2^) and pulsed-gradient SE echo planar with sensitivity encoding (acceleration factor = 2.5, TR = 8,986 ms, TE = 80 ms, 55 contiguous, 2.5 mm-thick axial slices, number of acquisitions = 2; acquisition matrix 96 × 96, with an in-plane pixel size of 1.87 × 1.87 mm and a FOV = 240 × 240 mm^2^) and diffusion gradients applied in 32 non-collinear directions using a gradient scheme which is standard on this system (gradient over-plus) and optimized to reduce echo time as much as possible. The b factor used was 1,000 s/mm. Fat saturation was performed to avoid chemical shift artifacts.

Using Scanner 2, the following brain MRI sequences were obtained: 3D T2-weighted [FOV = 256 × 256, pixel size = 1.21 × 1.21 mm, 192 slices, 1 mm thick, matrix = 256 × 256, TR = 5,500 ms, TE = 247 ms, inversion time (TI) 1–2 = 2,550- echo train length (ETL) = 173, acquisition time (TA) = 3.45 min]; sagittal 3D fluid-attenuation inversion recovery (FOV = 256 × 256, pixel size = 1 × 1 mm, 192 slices, 1 mm thick, matrix = 256 × 256, TR = 4,800 ms, TE = 270 ms, TI = 1,650 ms, ETL = 167, TA = 6.15 min); 3D high resolution T1-weighted turbo field echo. FOV = 256 × 256, pixel size = 1 × 1 mm, 204 slices, 1 mm thick, matrix = 256 × 256, TR = 7 ms, TE = 3.2 ms, TI = 1,000 ms, FA = 8, ETL = 240, TA = 8.53 min); and a diffusion-weighted sequence. FOV = 240 × 232 mm, pix = 2.14 × 2.69 mm, 56 slice, 2.3 mm thick, matrix = 112 × 85 TR = 5,900 ms, TE = 78 ms, 3 shells *b*-value = 700/1,000/2,855 s/mm^2^, along 6/30/60 non-collinear directions and 10 *b* = 0 volumes were acquired.

For acquisitions on both scanners, all slices were positioned to run parallel to a line that joins the most inferoanterior and inferoposterior parts of the corpus callosum.

### MRI analysis

#### Voxel-based morphometry

Voxel-based morphometry (VBM) was performed using SPM12 (http://www.fil.ion.ucl.ac.uk/spm/) and Diffeomorphic Anatomical Registration Exponentiated Lie Algebra (DARTEL) registration method (53) to investigate gray matter (GM) volume alterations, as described previously (54).

#### Gray matter volumes

GM maps of patients and healthy controls were parcellated into 90 Automated Anatomical Labeling (AAL) regions of interest to obtain regional GM volumes. Specifically, the AAL atlas was registered to the subjects' T1-weighted images using linear and non-linear registrations (FLIRT and FNIRT, respectively) (55, 56), as implemented in the FMRIB software library (FSL, http://www.fmrib.ox.ac.uk/fsl). Cortical GM maps were obtained from the segmentation step of VBM procedure (as described previously) (54), while maps of the basal ganglia (i.e., bilateral caudate, globus pallidus, putamen, and thalamus), hippocampus and amygdala were obtained using the FIRST tool in FSL (http://www.fmrib.ox.ac.uk/fsl/first/index.html). GM volumes were multiplied by the normalization factor derived from SIENAx (part of FSL; http://www.fmrib.ox.ac.uk/fsl/sienax/index.html) to correct for head size.

#### White matter tractography

DT MRI analysis was performed using the FMRIB Diffusion Toolbox in FSL (http://www.fmrib.ox.ac.uk/fsl/fdt/index.html) and the JIM6 software (Version 6.0, Xinapse Systems, Northants, UK, http://www.xinapse.com), as described previously (57). Maps of mean diffusivity (MD), fractional anisotropy (FA), axial diffusivity (axD) and radial diffusivity (radD) were obtained. Seeds for tractography of the corpus callosum (CC), corticospinal tract (CST), cingulate, inferior and superior longitudinal, and uncinate fasciculi were defined in the Montreal Neurological Institute (MNI) space on the FA template provided by FSL, as previously described (57, 58). The CC was segmented into three portions to identify the callosal fibers linking the precentral (CC-precentral), lateral premotor (CC-premotor) and supplementary motor areas (CC-supplementary motor), as previously described (59). Fiber tracking was performed in native DT MRI space using a probabilistic tractography algorithm implemented in FSL (probtrackx) (60). For each tract, the average MD, FA, axD and radD were calculated in the native space.

### Statistical analysis

Normal distribution assumption was checked by means of Q–Q plot and Shapiro-Wilks and Kolmogorov-Smirnov tests.

Sociodemographic and clinical features (i.e., age, sex, education, ALSFRS-r scores, disease duration, and progression rate) were compared between groups using ANOVA models or Pearson's chi square, as appropriate. Neuropsychological and MRI quantitative features (i.e., GM volumes and DT MRI metrics) were also compared between groups using separate ANOVA models, followed by *post-hoc* pairwise comparisons, Bonferroni-corrected for multiple comparisons and adjusted for age, sex, education, and—in the case of MRI variables—MR scanner. The threshold of statistical significance was set at *p* < 0.05. The SPSS Statistics 22.0 software was used.

VBM group comparisons were tested using ANCOVA model adjusting for total intracranial volume, age, sex, and MRI scanner type. Results were assessed at *p* < 0.05 Family-wise error (FWE)-corrected for multiple comparisons.

## Results

### Sociodemographic and clinical features

Table 1 summarizes the main sociodemographic and clinical variables of study groups. *TARDBP* and sMND patients were comparable in terms of sex, education, age at MRI and disease duration. Furthermore, patients were similar in terms of ALSFRS-r score and disease progression rate. Table 2 and Supplementary Table 1 report individual diagnoses and clinical features of included patients. The group of *TARDBP* patients included six men and five women with an age of onset ranging from 43 to 67 years old (59.8 ± 8.2). Their diagnoses included ALS (*n* = 7), PMA (*n* = 3), and PLS (*n* = 1). Nine out of 11 mutated patients presented with a limb onset of disease, with a tendency of having the right side involved first (6 out of 7 patients with a lateralized limb onset), although at the time of clinical evaluation muscle atrophy was mostly bilateral and symmetrical. Two patients with a limb onset were unable to date back a side of onset. Two patients had a bulbar onset (i.e., dysarthria and mild difficulties when swallowing). One of them (subject 5) had the fastest progression rate. At the time of MRI, ALSFRS-r was ranging from 20 to 44 (34.18 ± 8.83), considering that diagnosis was formulated from three up to 35 months after symptom onset. Disease progression rate was highly variable among mutated subjects (being faster in patients with bulbar presentation). No significant differences in disease progression velocity were recorded between sMND and mutated patients. Consistent with the fact that all MND phenotypes were represented, different combinations of upper and lower motor neuron signs were present at an individual basis (see Supplementary Table 1). No patients among *TARDBP* mutated and matched sMND fulfilled clinical criteria for behavioral or linguistic variants of FTD (57, 61, 62).

**Table 1:** Main clinical and demographic characteristics of subjects.

	**HC**	**sMND**	**TARDBP**	**p**
Age at MRI	58.99 ± 6.08 [47.63–72.70]	59.07 ± 7.68 [45.00–68.33]	59.78 ± 8.17 [44.14–70.95]	0.952
Sex (M/F)	11/11	6/5	6/5	0.958
Scanner type (S1/S2)	18/4	8/3	8/3	0.785
Education (years)	12.19 ± 3.34 [8–18]	12.73 ± 3.44 [6–17]	10.33 ± 3.39 [5–16]	0.265
Disease duration	–	63.0 ± 90.02 [8.00–277.00]	16.93 ± 17.61 [5.00–67.00]	0.113
ALSFRS-r	–	33.90 ± 6.37 [23–42]	34.18 ± 8.82 [20–44]	0.935
Disease progression rate	–	0.73 ± 0.74 [0.08–2.11]	1.40 ± 1.23 [0.16–4.00]	0.135

*Values are numbers or means ± standard deviations [range]. p-values refer to Pearson's chi square or ANOVA models (as appropriate)*.

*ALSFRS-r, revised version of the Amyotrophic Lateral Sclerosis Functional Rating Scale; F, female; HC, healthy controls; M, male; MND, motor neuron disease; S1/S2, Scanner 1/Scanner 2; sMND, sporadic MND. –, not applicable*.

**Table 2:** Individual socio-demographic and clinical features of MND patients.

**Subject**	**Mutation**	**Diagnosis**	**Sex (M/F)**	**Education (years)**	**Age at onset**	**Age at MRI (years)**	**Time onset to diagnosis (months)**	**ALSFSR-r [0-48]**	**MRC sum score**	**Disease progression rate**	**Bulb/limb onset**	**Site onset**	**Cognitive/** **behavioral profile**
1	*TARDBP*	ALS	M	8	70	71	7	44	NA	0.50	Limb	RUL	NA
2	*TARDBP*	ALS	F	8	61	63	13	44	NA	0.17	Limb	RLL	NA
3	*TARDBP*	ALS	F	11	50	56	35	36	103	0.18	Limb	LL	ALS-ci
4	*TARDBP*	PMA	M	8	62	62	3	33	79	2.50	limb	UL	PMA-ci
5	*TARDBP*	ALS	F	11	48	48	4	20	91	4.00	Bulb	–	ALS-ci
6	*TARDBP*	PMA	M	5	60	61	10	38	91	0.83	Limb	RUL	NA
7	*TARDBP*	ALS	M	NA	62	63	8	20	NA	2.80	Limb	LUL	NA
8	*TARDBP*	PLS	F	13	55	56	9	35	NA	1.08	Limb	RLL	Normal
9	*TARDBP*	ALS	F	16	67	67	4	40	95	1.60	Bulb	–	Normal
10	*TARDBP*	PMA	M	NA	65	67	11	25	50	1.43	Limb	RLL	PMA-ci
11	*TARDBP*	ALS	M	13	43	44	18	41	NA	0.36	Limb	RUL	NA
12	–	PMA	M	16	54	60	11	33	78	0.21	Limb	RUL	Normal
13	–	ALS	F	17	65	67	19	40	91	0.36	Limb	LL	Normal
14	–	ALS	F	13	57	58	8	33	91	1.36	Limb	NA	NA
15	–	PMA	M	13	59	60	6	32	109	2.00	Limb	LUL	NA
16	–	ALS	M	6	65	66	6	29	111	2.11	Bulb	–	Normal
17	–	PMA	M	13	41	65	156	25	66	0.08	Limb	RLL	NA
18	–	ALS	F	17	50	54	12	38	111	0.25	Bulb	–	Normal
19	–	ALS	F	11	40	45	25	23	60	0.38	Limb	LL	Normal
20	–	ALS	F	8	58	59	15	42	105	0.40	Limb	NA	NA
21	–	ALS	M	13	67	68	13	41	107	0.55	Limb	NA	NA
22	–	ALS	M	13	45	47	36	37	106	0.31	Limb	NA	NA

*ALS, amyotrophic lateral sclerosis; -ci, cognitive impairment; F, female; LL, lower limbs; LUL, center upper limb; M, male; MRC, Medical Research Council scale; NA, not available; PMA, primary muscular atrophy; PLS, primary lateral sclerosis; RLL, right lower limb; RUL, right upper limb; UL, upper limbs. –, not applicable*.

### Neuropsychological features

Even if data for a cognitive/behavioral diagnosis according to the revised Strong criteria (51) were available for only about half of our cohort, due to the fact that most patients here included were enrolled before 2017, 5/5 sMND patients presented with a normal cognitive profile, while 4/6 mutated patients showed signs of mild cognitive impairment (51). Table 3 reports the neuropsychological test scores of sMND and *TARDBP* mutated patients. In terms of global cognition, the two groups of patients scored similar results at MMSE. Furthermore, both groups were comparable in terms of memory, executive functions, visuospatial abilities and fluency. Of note, visuospatial abilities were only tested with Rey figure copy and data from the sMND population were available for only one subject. Language was evaluated with the Battery for aphasic deficit analysis (BADA). At the action naming subtest of BADA, *TARBDP* showed a lower performance compared to controls (*p* = 0.003) and sMND (*p* = 0.019). At the noun naming subtest of BADA, *TARDBP* mutation carriers performed poorer than controls and sMND, although pairwise comparisons did not reach statistical significance (*p* = 0.081 and *p* = 0.088, respectively).

**Table 3:** Neuropsychological features of healthy controls and MND patients.

	**Healthy controls**	**sMND**	***TARDBP*** **MND**	***p***
*N* (total sample)	22	11	11	
**Global cognition**				
MMSE	29.33 ± 0.81 [28–30]	28.22 ± 0.97 [27–30]	28.00 ± 3.06 [20–30]	0.157
	(22)	(9)	(10)	
**Memory**				
Digit span forward	6.33 ± 1.13 [4–9]	6.13 ± 1.64 [4–9]	5.44 ± 1.24 [3–7]	0.361
	(15)	(8)	(9)	
RAVLT delayed	9.00 ± 3.48 [3–15]	9.75 ± 3.81 [4–15]	7.22 ± 2.64 [4–13]	0.274
	(15)	(8)	(9)	
RAVLT recognition	14.23 ± 1.01 [12–15]	14.40 ± 0.89 [13–15]	14.29 ± 0.95 [13–15]	0.960
	(13)	(5)	(7)	
**Executive functions**				
CPM	30.92 ± 3.66 [22–35]	30.43 ± 5.11 [21–36] (7)	28.29 ± 6.31 [16–34]	0.775
	(13)		(7)	
Digit span backward	4.60 ± 0.91 [3–6]	4.75 ± 1.17 [3–6]	4.33 ± 1.23 [3–6]	0.783
	(15)	(8)	(9)	
**Visuospatial abilities**				
Rey figure copy	31.60 ± 2.30 [29–35]	30.67 ± 2.08 [29–33]	30.13 ± 6.25 [21.5–36]	0.762
	(4)	(3)	(4)	
**Language**				
BADA (noun)	29.86 ± 0.38 [29, 30]	29.67 ± 0.81 [28–30]	28.71 ± 1.38 [27–30]	**0.039**
	(7)	(6)	(7)	
BADA (action)	28.00 ± 0.00 [28]	27.67 ± 0.51 [27, 28]	24.86 ± 2.34 [21–27]	**0.002**
	(7)	(6)	(7)^*^$	
Token test	32.70 ± 2.14 [30–35]	33.70 ± 1.50 [33–36]	33.25 ± 1.26 [32–35]	0.451
	(6)	(4)	(4)	
**Fluency**				
Index PF^***^	4.79 ± 2.35 [3.28–12.05]	6.07 ± 4.04 [1.90–13.50]	9.54 ± 8.95 [2.72–31.00]	0.181
	(13)	(8)	(8)	
Index SF^***^	3.63 ± 0.81 [2.49–5.53]	4.15 ± 1.93 [2.20–8.04]	10.59 ± 15.99 [3.49–50.00]	0.244
	(13)	(7)	(8)	
**Mood & Behavior**				
FBI total		1.25 ± 0.96 [0–2]	3.38 ± 4.27 [0–13]	0.234
		(4)	(7)	

*Values are numbers or means ± standard deviations [range] (N of subjects). P-values refer to ANOVA models, followed by Bonferroni correction for multiple comparisons adjusted for age, sex and education*.

*BADA, battery for aphasic deficit analysis; CPM, colored progressive matrices; FBI, Frontal Behavioral Inventory; PF, phonemic fluency; SF, semantic fluency; sMND, sporadic motor neuron disease*.

* *significantly different from healthy controls;*

*$, significantly different from sMND;*

*** *verbal fluency indices were obtained as following: time for generation condition—time for control condition (reading or writing generated words)/total number of items generated. Bold indicates significant values*.

### MRI results

#### Voxel-based morphometry

**Figure 1:**
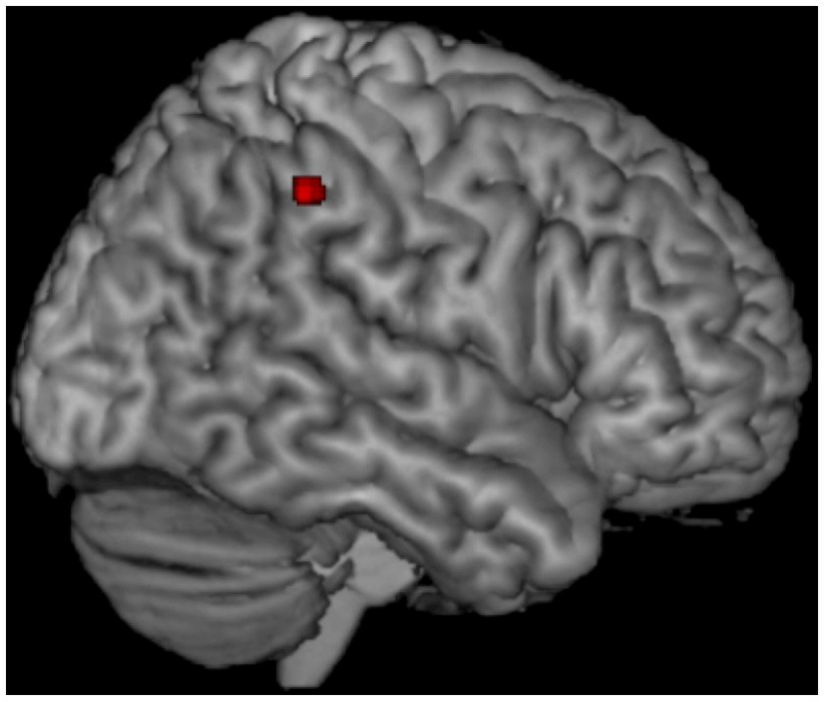
Voxel-based morphometry (VBM) results. Voxel-based analysis showing gray matter (GM) volume loss in *TARDBP* MND patients relative to healthy controls. Results are overlaid on a three-dimensional rendering of the Montreal Neurological Institute standard brain and displayed at *p* < 0.05 corrected for multiple comparisons. Only clusters comprising at least 20 contiguous voxels are shown. Analysis is corrected for intracranial volume, age, sex, and MR scanner type.

Figure 1 reports results of VBM for regions that survived a *p* < 0.05 FWE, corrected at cluster level. Compared with controls, *TARDBP* patients showed GM atrophy at the level of the right lateral parietal cortical regions, including the supramarginal and angular gyri (cluster size = 49; MNI coordinates of peak of significance: x = 60, y = −40, z = 45; *T*-value = 5.83).

#### GM volumes

**Figure 2:**
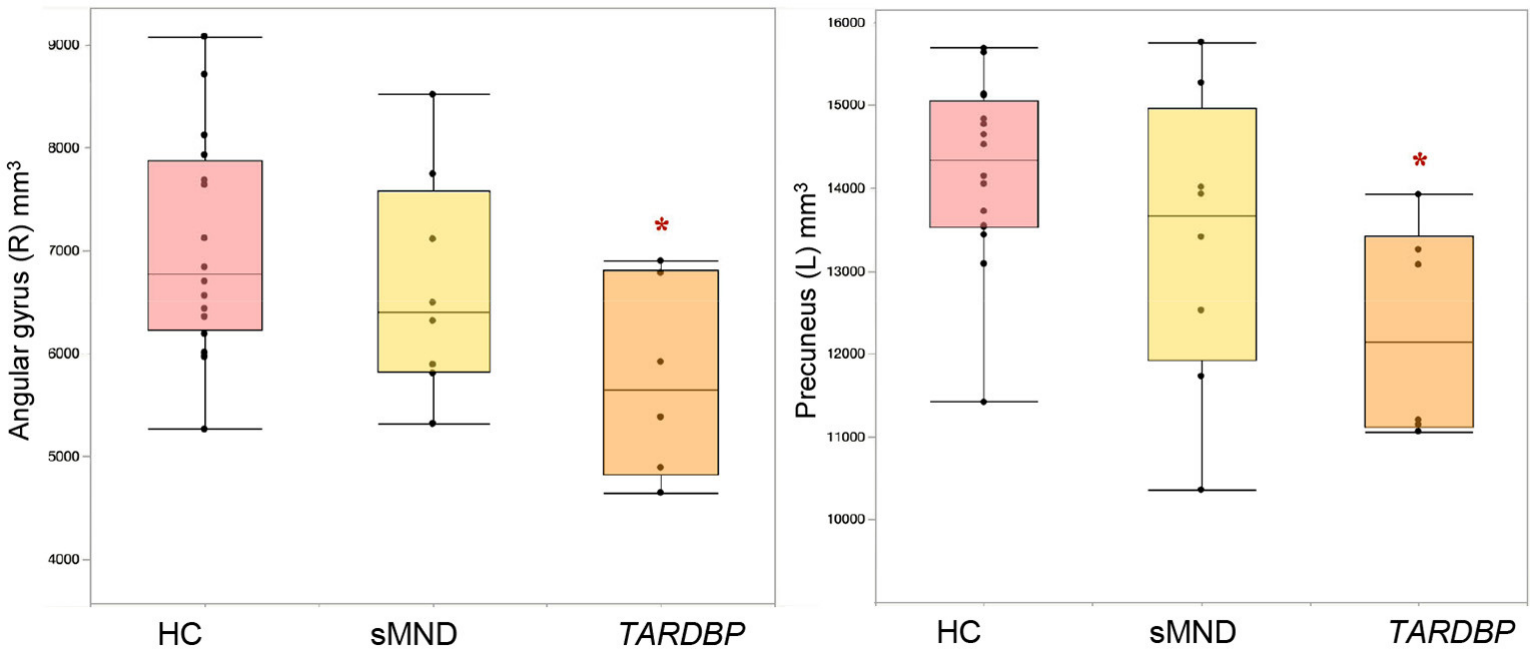
Gray matter (GM) cortical volumes showing significant differences in MND patients and controls. Values refer to mm^3^. Comparisons between groups were performed using age-, sex-, and MRI scanner-adjusted analysis of variance models, followed by *post-hoc* pairwise comparisons, Bonferroni-corrected for multiple comparisons. HC, healthy controls; L, left; R, right; sMND, sporadic motor neuron disease. *Significantly different from HC.

Figure 2 and Supplementary Table 2 summarize GM volume comparisons. A significant GM volume reduction was found in the left precuneus (*p* = 0.002) and right angular gyrus of *TARDBP* patients (*p* = 0.037), compared to controls. No other significant results emerged in our analysis, although a trend toward a greater atrophy of the left angular gyrus was also observed in *TARDBP* patients, compared with controls (*p* = 0.08).

#### DT MRI

**Figure 3:**
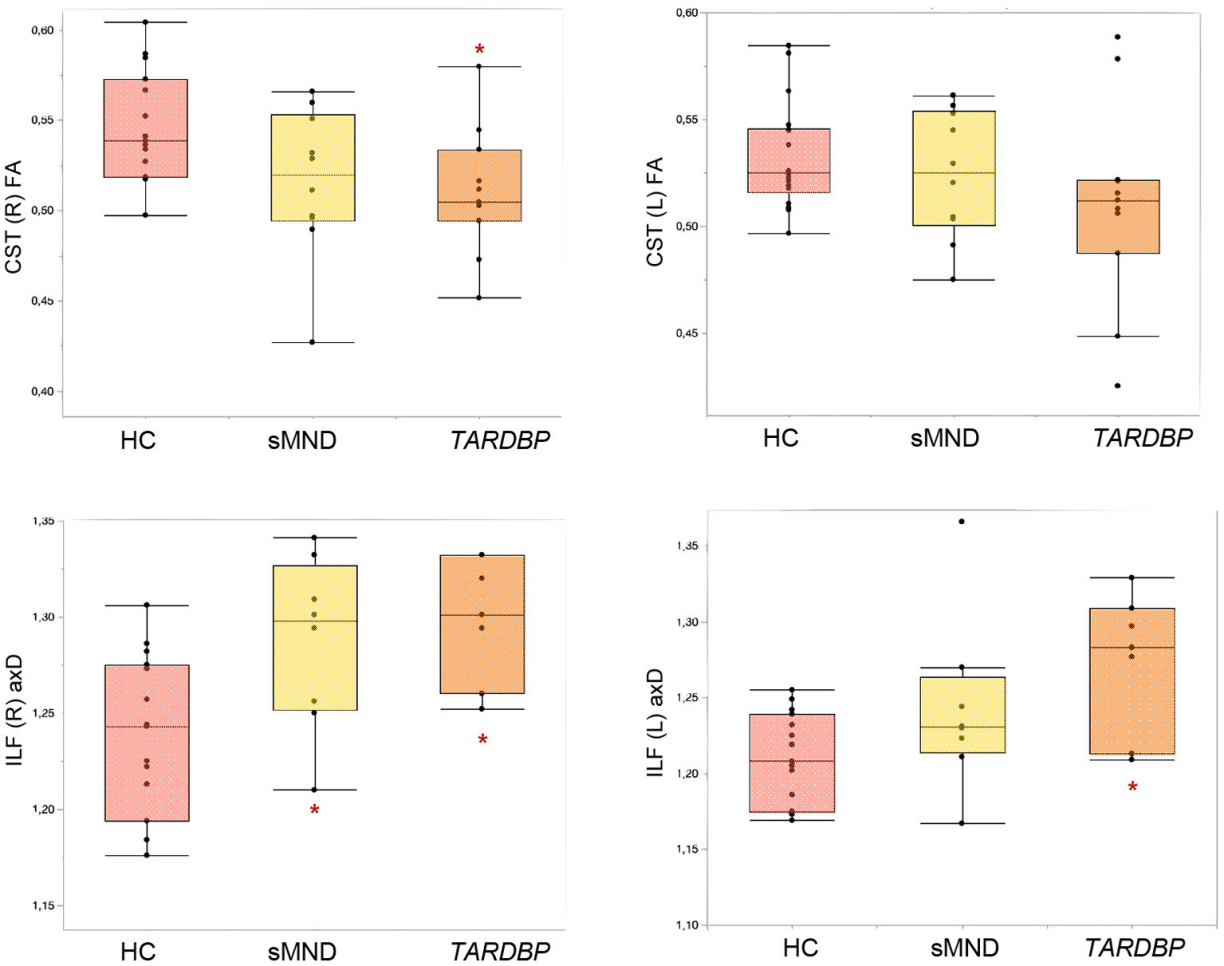
Diffusion tensor (DT) MRI metrics of white matter (WM) tracts showing significant differences in MND patients and controls. Comparisons between groups were performed using age-, sex-, and MRI scanner-adjusted analysis of variance models, followed by *post hoc* pairwise comparisons, Bonferroni-corrected for multiple comparisons. CST, corticospinal tract; HC, healthy controls; ILF, inferior longitudinal fasciculus; L, left; R, right; sMND, sporadic motor neuron disease. *Significantly different from HC.

As shown in Figure 3 and Supplementary Table 3, the right CST showed decreased fractional anisotropy (FA) only in *TARDBP* patients, compared to controls (*p* = 0.035). The left inferior longitudinal fasciculus (ILF) showed higher values of axial diffusivity (axD) in *TARDBP* compared to controls (0.017); the right ILF instead showed increased axD both in sMND compared to controls (*p* = 0.047) and in *TARDBP* cases compared to controls (*p* = 0.019).

## Discussion

To the best of our knowledge, this was the first study analyzing a relatively large cohort of *TARDBP* mutated MND patients with quantitative MRI advanced techniques. Previous studies have described single cases or small case series of families or unrelated subjects affected by *TARDBP* mutations causing a syndrome of the FTD-ALS spectrum (14–22, 33). However, none of these reports involved the use of advanced MRI techniques. Available data in the literature only included description of cortical atrophy by visual inspection of conventional MRI sequences, without displaying quantitative information on GM volumes or any information at all on WM alterations of patients affected by *TARDBP* mutations. We have previously described GM atrophy in a cohort of genetic FTLD (32), which included some of the *TARDBP* patients included here. However, in the present study, we expanded our cohort and performed a systematic analysis exploring WM features at DT MRI as well. Our study provides therefore a thorough, combined picture of GM and WM structural alterations in patients carrying this mutation, highlighting neuroanatomical differences compared to clinically matched sMND cases.

In the present cohort, all patients carrying mutations in the *TARDBP* gene presented with a pure MND phenotype, as no patient fulfilled established clinical criteria for a defined FTD syndrome (61, 62). The clinical presentation was heterogeneous, with patients presenting with variable combinations of upper and lower motor neuron signs, configuring all the main MND phenotypes, including ALS, PMA and PLS. Furthermore, mutated patients presented with either a limb or a bulbar onset (in one case, with a very fast disease progression). Therefore, in our cohort, no specific motor phenotype was associated with a *TARDBP* mutation. The right-sided onset of motor symptoms reported by most TARDBP patients with a limb onset might have been influenced by the fact that all patients were right-handed, as at time of neurological evaluation there was no clear consistent lateralization of clinical findings. Such heterogeneity diverges from previous reports, in which *TARDBP* mutations with MND have been mostly associated with an ALS syndrome, with the exception of one case of PMA (63), in a patient whose first symptom was camptocormia. All other cases reported an ALS presentation (14–22, 33), with variable associations of FTD phenotypes (of note, often consistent with temporal variant of FTD or semantic dementia) (16).

When examining neuropsychological data, sMND and *TARDBP*-mutated MND patients were comparable in terms of global cognitive measures, as well as performance in most cognitive domains and behavioral symptoms. However, *TARDBP* patients differed from controls and matched sMND patients in terms of linguistic performance. Indeed, at the action and noun naming subtests of a battery examining comprehension, denomination and repetition of nouns and verbs, mutated patients performed significantly worse than sMND. Even considering the limitation that sufficiently complete neuropsychological data for a formal cognitive diagnosis (51) was not available for all subjects, four mutated patients fulfilled Strong criteria for MND with cognitive impairment (51), suggesting a more globally distributed, although mild, damage to cognitive functions compared with the sMND sample, matched for all other clinical features. This is consistent with data reported in the literature, in which there are cases of *TARDBP* patients presenting with (or developing in the course of the disease) cognitive impairment, particularly in the executive and linguistic domains (15, 16). However, further data are warranted to confirm this as a characteristic feature of *TARDBP* mutated patients presenting with isolated MND.

Compared with controls, VBM showed a circumscribed, distinctive atrophy of the right lateral parietal cortex in MND patients with a *TARDBP* mutation, confirmed by GM volumetric reduction of the right angular gyrus. Moreover, GM volumetric analysis also demonstrated significant atrophy of the left precuneus, as well as an almost significant trend for the left angular gyrus in *TARDBP* mutation carriers. The lateral parietal cortex and the precuneus are known to be cross-modal hubs where multisensory information converging from frontal and temporal inputs is processed and integrated (64, 65). Among lateral parietal cortical regions involved, the key region showing atrophy only in mutation carriers was the angular gyrus. This area has been implied in higher language abilities, and its activation has been registered during semantic processing (66) and semantic tasks on auditory (67) and visual (68) stimuli. A recent report has specifically related hypoperfusion of the angular gyrus with language deficits in ALS (57, 69). With the limitations of the small number of patients included in our study, which could not allow us to properly run a correlation analysis, atrophy of these cortical regions could possibly justify, at least in part, the lower scores obtained at language assessments by mutated patients.

Previous studies have described cortical atrophy in more anterior regions in sMND patients, such as the primary motor and premotor cortices, as well as prefrontal and temporal regions (32, 70, 71). A more posterior pattern of atrophy has instead been associated with other genetic forms of MND—mostly, related to *C9orf72* pathologic expansions (32, 72, 73)—, leading to the hypothesis that genetic mutations may promote neurodegeneration also in areas that are not typically involved in MND, possibly due to an accelerated neurodegenerative process (32, 72, 73).

As previously highlighted, the present study was the first to assess WM damage using DT MRI in *TARDBP* mutated patients in a systematic way. Based on our results, *TARDBP* patients and sMND shared a significant microstructural damage of the CST and ILF, although mutation carriers showed a slightly more extensive involvement, in comparison with the matched sMND sample. DT MRI of the CST is a well-known quantitative measure of upper motor neuron damage in MND (74, 75), whereas the ILF (connecting occipital and temporal lobes) is known to be involved not only in spatial processing but also in language processes (76–78). It has been suggested that the ILF acts in the interplay of the semantic-ventral stream (79–84), as supported by consistent evidence obtained from patients with semantic dementia, in which the ILF is highly disrupted (58, 85). Of note, a significant proportion of FTD patients with a *TARDBP* mutation were found to fulfill criteria for semantic dementia by other reports (16). So far, previous studies on ALS patients have demonstrated a correlation between alterations of the ILF and emotional processing disorders (86, 87), but future investigation will be needed to perfect our knowledge on the role of this WM tract for linguistic impairment in pure MND.

This study is not without limitations. First, to reach the greatest number of *TARDBP* patients, we had to include subjects who performed MRI on two different scanners. This limitation was partially overcome by including scanner type as a covariate in our statistical analyses. Secondly, accurate cognitive/behavioral phenotyping was not available for the whole cohort of patients, thus not allowing a proper correlation analysis between neuroanatomical and cognitive data in a sufficiently powered sample. Moreover, we did not have systematic information regarding mutations or variants of unknown significance on less common MND-related genes, other than those analyzed with Sanger sequencing (e.g., those related with hereditary spastic paraplegia, etc.). Although we could not completely exclude the presence of these alterations in the sMND sample, we still could describe the *distinctive* neuroanatomical alterations of the *TARDBP* mutated subjects, as per declared objective of the present study. One last pitfall of this study is its cross-sectional design, as a longitudinal approach would have allowed to keep track of cortical and subcortical damage as the disease unfolds and different symptoms come at play. Longitudinal studies are warranted to better understand the clinical relevance of these findings in mutated subjects, in order to identify useful outcome measures in future gene-targeting clinical trials.

In conclusion, our findings suggest that MND patients carrying a *TARDBP* may present with a heterogeneous clinical phenotype. However, we suggest that a distinctive, mild impairment of the linguistic domains, together with a prominent damage to parietal GM structures might be the hallmark of this uncommon, but significant cause of genetically-determined MND.

## Data availability statement

The raw data supporting the conclusions of this article will be made available by the authors, without undue reservation.

## Ethics statement

The studies involving human participants were reviewed and approved by IRCCS Ospedale San Raffaele. The patients/participants provided their written informed consent to participate in this study.

## Author contributions

ES, AG, MF, and FA conceived and designed the study. ES, AG, NR, EC, VC, VS, and AC acquired data. ES, AG, EC, VC, TD, and LP analyzed data. All authors contributed to the article, drafted the manuscript, and approved the submitted version.

## Funding

This work was supported by the Italian Ministry of Health (GR-2011-02351217, GR-2013-02357415, and RF-2011-02351193) and AriSLA (ConnectALS), European Research Council (StG-2016_714388_NeuroTRACK).

## Conflict of Interest

The authors declare that the research was conducted in the absence of any commercial or financial relationships that could be construed as a potential conflict of interest.

## Publisher's note

All claims expressed in this article are solely those of the authors and do not necessarily represent those of their affiliated organizations, or those of the publisher, the editors and the reviewers. Any product that may be evaluated in this article, or claim that may be made by its manufacturer, is not guaranteed or endorsed by the publisher.
