# Supplementary material for: Profiling morphologic MRI features of motor neuron disease caused by TARDBP mutations
Source: Front Neurol. 2022 Jul 15;13:931006. doi: 10.3389/fneur.2022.931006 (PMC9334911; doi:10.3389/fneur.2022.931006)
Supplement: Supplementary file 1 [file Data_Sheet_1.docx]

**Supplementary material**

**Supplementary Table 1.** Detailed findings at neurological examination in ALS patients.

|  | Mutation | Diagnosis | Increased reflexes | | | Spasticity | | Muscular atrophy | Extensor plantar reflex |
| --- | --- | --- | --- | --- | --- | --- | --- | --- | --- |
|  |  |  | Bulbar | UL | LL | UL | LL |  |  |
| Subject 1 | *TARDBP* | ALS | - | + | - | - | - | UL | - |
| Subject 2 | *TARDBP* | ALS | - | - | - | - | - | LU left | - |
| Subject 3 | *TARDBP* | ALS | + | + | + | + | + | UL (distal) | + |
| Subject 4 | *TARDBP* | PMA | - | - | - | - | - | UL, LL | - |
| Subject 5 | *TARDBP* | ALS | + | + | - | - | - | UL, LL | + |
| Subject 6 | *TARDBP* | PMA | - | - | - | - | - | UL | - |
| Subject 7 | *TARDBP* | ALS | + | + | + | - | - | UL, LL | + |
| Subject 8 | *TARDBP* | PLS | + | + | + | - | ± | UL, LL | + |
| Subject 9 | *TARDBP* | ALS | - | - | + | - | + | None | - |
| Subject 10 | *TARDBP* | PMA | - | - | - | - | - | UL, LL | - |
| Subject 11 | *TARDBP* | ALS | - | + | - | - | - | LL | - |
| Subject 12 |  | PMA | - | - | - | - | - | UL | - |
| Subject 13 |  | ALS | - | - | + | - | - | UL (distal) | - |
| Subject 14 |  | ALS | + | + | + | ++ | ++ | UL, LL | - |
| Subject 15 |  | PMA | - | - | - | - | - | UL, LL | - |
| Subject 16 |  | ALS | + + | + | + | + | + | UL left | + |
| Subject 17 |  | PMA | - | - | - | - | - | UL, LL | - |
| Subject 18 |  | ALS | + | + | + | - | - | UL, LL | + |
| Subject 19 |  | ALS | ++ | + | + | - | - | bulbar, UL, LL | + |
| Subject 20 |  | ALS | + | - | - | - | - | UL (distal) | + |
| Subject 21 |  | ALS | - | - | + | - | - | UL (distal), LL | - |
| Subject 22 |  | ALS | - | + | ++ | + | + | UL (distal) | - |

*Abbreviations: ALS= amyotrophic lateral sclerosis; LL= lower limbs; PMA= progressive muscular atrophy; PLS= primary lateral sclerosis; UL= upper limbs*

**Supplementary Table 2.** GM volumes (mm^3^) of the 90 Automated Anatomical Labeling (AAL) regions in healthy controls and MND patients.

|  | **Healthy controls** | **sMND** | ***TARDBP* MND** | **p** |
| --- | --- | --- | --- | --- |
| Precentral (L) | 8581.01 ± 1206.48 (6558.84 – 10914.03) | 8912.88 ± 1901.75 (6534.03 – 11155.79) | 8941.73 ± 1871.87 (7099.43 – 12359.28) | 0.823 |
| Precentral (R) | 8175.82 ± 1064.70 (6655.29 – 1038184) | 8697.51 ± 1226.52 (6944.52 – 10874.83) | 8897.97 ± 972.71 (7519.91 – 9988.57) | 0.307 |
| Frontal superior (R) | 11898.12 ± 1681.38  (8493.35 – 15077) | 12249.74 ± 1674.89 (9992.57 - 14443.09) | 11702.11 ± 400.10  (11091.42 - 12181.05) | 0.747 |
| Frontal superior (L) | 12181.05 ±  1288.39 (8609.38 - 13407.98) | 11225.13 ± 1567.67  (8713.31 - 13343.81) | 10878.44 ± 1495.73  (9199.48 - 13402.14) | 0.715 |
| Frontal superior orbital (L) | 3763.68 ± 514.01  (2793.10 - 4481.40) | 4016.33 ±  679.67 (3101.65 - 5128.55 | 4015.05 ± 397.17  (3252.99 - 4345.94 | 0.405 |
| Frontal superior orbital (R) | 4004.44 ± 539.98  (3163.90 - 5120.87) | 4155.19 ± 697.85 (3280.37 - 5170.50) | 4134.04 ± 479.08 (3433.06 -  4706.12) | 0.592 |
| Frontal Middle (L) | 16430.17± 2073.72 (13343.00 - 22003.69) | 16330.94  ± 3104.03 (11986.32 -  21070.66) | 15814.66 ± 1701.53 (12566.49 - 17302.18) | 0.737 |
| Frontal middle (R) | 19340.66 ± 2323.91 (14942.73 -  23907.64) | 18528.31 ± 2273.28 (15335.71 - 22432.80) | 18566.48 ± 2326.16 (15771.46 -  22220.42) | 0.569 |
| Frontal middle orbital (L) | 3507.36 ± 411.15 (2686.25 - 4151.91) | 3673.44 ± 550.55 (2729.73 -4316.37) | 3660.62 ± 729.48 (2981.03 - 5045.04) | 0.722 |
| Frontal middle orbital (R) | 4117.03 ± 739.20 (2681.15 - 5110.47) | 4307.00 ± 720.06  (3375.10 -5405.00) | 4109.53 ± 434.56 (3279.89 - 4443.38) | 0.683 |
| Frontal inferior opercular (L) | 3067.38 ± 411.05 (  2216.33 - 3927.36) | 3454.12 ± 745.67 (2372.46 - 4738.67) | 3440.28 ± 780.30 (2571.05 - 4771.78) | 0.296 |
| Frontal inferior opercular (R) | 5165.06 ± 674.14 (4108.38 -  6678.93) | 5154.33 ± 944.09 (3606.05 -  6312.51) | 5497.72 ±  626.34 (4515.75 -  6356.20) | 0.710 |
| Frontal inferior triangular (L) | 8289.08 ± 1481.56 (6564.21 - 10916.68) | 8523.89 ± 1933.40 (6458.40 - 11387.68) | 9355.85 ± 1394.29  (8112.43 - 11755.27) | 0.516 |
| Frontal inferior triangular (R) | 6930.44 ±  896.49 (5403.44 -  8369.82) | 7118.12 ±  2127.56 (4643.60 - 11566.38) | 7305.03 ± 1601.50 (4219.41 - 8649.39) | 0.941 |
| Frontal inferior orbital (L) | 7579.39 ±  842.14 (6111.50 -  9281.46) | 7983.18 ± 885.50 (6597.63 - 9437.31) | 7770.54 ± 696.23 (6895.76 - 8611.72) | 0.375 |
| Frontal inferior orbital (R) | 6356.27 ± 1132.10 (4666.18 - 8995.21) | 6404.03 ± 1284.50 (4395.03 - 7757.90) | 5911.21 ± 724.36 (5052.02 - 6925.25) | 0.627 |
| Rolandic opercular (L) | 4011.75 ± 385.93  (3204.61 - 4600.66) | 4217.18 ±  972.24 (3202.70 -  6104.11) | 4277.85 ±  343.29 (3799.32 -  4728.93) | 0.538 |
| Rolandic opercular (R) | 5711.74 ±  656.16 (4714.35 -  6897.77) | 5981.42 ±  852.39 (4839.60 -  7106.34) | 5790.10 ± 527.85 (4886.53 - 6311.95) | 0.515 |
| Supplementary motor area (L) | 7355.55 ± 999.41 (5982.87 – 9516.14) | 7174.01 ± 1632.53 (4753.91 – 8909.20) | 7154.89 ± 1459.99 (5779.66 – 9491.62) | 0.845 |
| Supplementary motor area (R) | 7103.46 ± 1048.18 (5365.87 – 8971.48) | 6729.10 ± 1179.92 (4526.41 – 7985.52) | 7478.47 ± 1551.30 (5778.81 – 9752.57) | 0.611 |
| Olfactory (L) | 1522.93 ± 203.59 (1214.13 - 1948.56) | 1595.08 ± 223.38 (1290.95 - 1930.63) | 1491.36 ± 121.83 (  1269.36 - 1639.61) | 0.524 |
| Olfactory (R) | 1536.49 ± 184.29 (1299.52 - 1826.58) | 1525.79 ±  155.47 (1270.19 -  1710.76) | 1492.18 ±  199.19 (1349.55 -  1889.50) | 0.845 |
| Frontal superior medial (L) | 9749.42 ± 1380.68 (7770.25 - 12207.07) | 9805.68 ± 1303.32 (7826.15 - 11526.02) | 10260.61 ± 1027.36 (8625.73 - 11408.22) | 0.784 |
| Frontal superior medial (R) | 8901.08 ± 864.22 (7197.91 - 10084.26) | 8591.98 ± 859.71 (7694.09 -10281.77) | 9259.92 ± 954.53 (8271.30 - 10948.17) | 0.429 |
| Frontal middle orbital (L) | 2859.65 ±  536.93 (2109.97 -  3939.07) | 3079.35 ±  675.38 (2050.92 -  4106.66) | 2915.70 ± 541.96 (2446.59 -  3948.72) | 0.348 |
| Frontal middle orbital (R) | 3744.09 ± 566.41 (2788.12 - 4581.77) | 4048.74 ± 840.83 (2336.50 -5164.78) | 3589.09 ± 553.76 (3041.22 -4481.43) | 0.145 |
| Rectus (L) | 3208.42 ±  396.93 (2506.44 -  3919.63) | 3505.63 ±  601.77 (2764.53 -  4484.15) | 3319.88 ±  328.26 (2996.50 -  3825.66) | 0.099 |
| Rectus (R) | 3284.45 ±  336.93 (2629.80 -  4000.49) | 3488.03 ± 573.69 (2998.12 - 4665.27) | 3442.68 ± 393.30 (3129.68 - 4149.56) | 0.079 |
| Insula (L) | 10556.17 ±  918.98 (8896.28 - 12271.15) | 10458.33 ±  1097.26 (8965.27 -12420.31) | 10462.72 ±  598.45 (9569.46 -  11221.92) | 0.831 |
| Insula (R) | 10002.65 ± 1070.07 (7848.19 - 11964.76) | 9683.51 ± 850.93 (8765.98 -10859.58) | 10174.62 ±  784.05 (9003.72 - 11230.50) | 0.667 |
| Cingulum anterior (L) | 6275.94 ± 781.19 (5068.51 - 7717.41) | 5828.61 ± 855.45 (4622.53 - 7173.37) | 5674.35 ±  740.69 (4736.37 - 6530.88) | 0.145 |
| Cingulum anterior (R) | 6209.56 ± 914.86 (4590.81 - 7496.42) | 5736.11 ± 619.56 (5021.51 - 6989.76) | 5528.63 ± 1232.20 (3751.81 - 6796.17) | 0.213 |
| Cingulum middle (L) | 8455.17 ± 1202.87 (6136.69 - 11098.45) | 8667.43 ± 1004.20 (7466.58 - 10151.30) | 8679.90 ± 575.25 (7515.31 - 9057.04) | 0.787 |
| Cingulum middle (R) | 9348.86 ± 9348.86 (7374.59 - 11435.33) | 9825.16 ± 1373.58 (7893.91 - 12165.55) | 9247.33 ± 495.44 (8902.68 - 10237.96) | 0.230 |
| Cingulum posterior (L) | 1546.49 ± 232.20 (1089.57 -1919.43) | 1574.71 ± 232.78 (1238.28 - 1808.59) | 1407.02 ± 226.67 (1062.92 - 1682.10) | 0.278 |
| Cingulum posterior (R) | 799.15 ± 117.45 (573.61 - 1004.72) | 793.49 ± 114.06 (656.79 - 975.36) | 712.29 ± 139.05 (505.01 - 842.70) | 0.298 |
| Hippocampus (L) | 5061.58 ± 482.02 (4311.09 - 6027.16) | 5252.66 ± 293.56 (4843.50 -  5630.07) | 5408.09 ± 239.99 (5068.07 -  5666.33) | 0.141 |
| Hippocampus (R) | 5042.22 ± 539.31 (4019.49 - 6176.73) | 5149.51 ± 244.86  (4729.67 - 5447.76) | 5249.73 ± 405.87 (4735.38 - 5942.78) | 0.652 |
| Parahippocampal (L) | 4908.71 ± 275.97 (4528.76 - 5515.68) | 5152.49 ± 345.74 (4696.52 - 5674.77) | 5064.58 ± 642.67 (4365.65 - 6207.05) | 0.332 |
| Parahippocampal (R) | 5559.82 ± 360.24 (4988.86 - 6332.95) | 5735.50 ± 305.51 (5625.89 - 6609.93) | 5862.07 ±  316.73 (5360.30 -  6191.06) | 0.064 |
| Amygdala (L) | 1259.67 ± 135.69 (1068.18 - 1500.84) | 1378.70 ± 89.42 (1189.80 - 1510.55) | 1253.22 ±  90.03 (1119.22 -  1374.58) | 0.077 |
| Amygdala (R) | 1314.09 ± 141.09 (1062.82 - 1578.38) | 1363.08 ± 79.74 (1259.47 -1493.76) | 1307.98 ± 88.11 (  1186.61 - 1427.74) | 0.305 |
| Calcarine (L) | 10482.02 ± 1487.82 (8081.00 - 12741.46) | 9528.14 ± 1332.05 (7673.33 - 12028.28) | 9750.06 ± 2138.77 (6744.58 - 12065.00) | 0.427 |
| Calcarine (R) | 5882.24 ±  1121.36 (4324.78 -  8236.86) | 946.91 ± 334.78 (4267.80 - 7580.35) | 826.95 ± 337.60 (463749 - 6758.00) | 0.962 |
| Cuneus (L) | 6040.34 ± 876.78 (5010.83 -  8222.43) | 5870.59 ± 863.66 (4747.61 - 7078.71) | 5481.63 ± 748.54 (4192.94 -  6377.53) | 0.518 |
| Cuneus (R) | 4225.20 ± 589.53 (3172.46 -  5389.28) | 765.94 ±  270.80 (3327.39 -  5640.38) | 4054.45 ± 524.80 (3280.07 - 4773.45) | 0.555 |
| Lingual (L) | 10242.74 ± 1348.40 (7159.18 -  12453.72) | 9925.91 ± 957.59 (8391.78 -  11577.38) | 9534.02 ±  1442.53 (7800.70 -  11315.36) | 0.426 |
| Lingual (R) | 7521.84 ± 822.45 (5803.84 -  8707.77) | 8006.08 ±  1074.52 (6752.94 - 9883.43) | 7294.02 ± 1447.84 (5118.52 - 11315.36) | 0.291 |
| Occipital superior (L) | 3604.14 ± 632.88 (2297.61 - 4894.81) | 3435.80 ± 811.43 (2575.35 - 4644.30) | 3348.54 ± 471.53 (2633.42 - 3936.38) | 0.648 |
| Occipital superior (R) | 4021.96 ± 838.59 (2657.64 - 5624.88) | 4216.51 ± 654.86 (3208.03 - 4938.98) | 3574.90 ± 801.13 (2495.28 - 4538.32) | 0.395 |
| Occipital middle (L) | 13588.09 ± 1638.45 (11167.75 - 16313.73) | 13492.54 ± 1787.56 (11272.66 - 16907.91) | 13398.81 ± 1843.82 (10928.22 -  15698.15) | 0.929 |
| Occipital middle (R) | 8864.90 ±  1486.82 (6496.97 - 11908.29) | 9563.34 ± 974.54 (8564.50 - 11261.34) | 8651.41 ± 1451.65 (6486.55 - 9740.06) | 0.315 |
| Occipital inferior (L) | 3820.27 ±  663.75 (2923.71 -  5603.37) | 3562.19 ±  504.03 (2575.06 -  4001.42) | 3326.04 ± 488.85 (2388.36 - 3705.72) | 0.183 |
| Occipital inferior (R) | 4095.53 ± 751.63 (3119.31 - 6086.89) | 3888.47 ± 3888.47 (2557.42 - 4924.56) | 3388.73 ± 665.35 (2316.80 - 4291.89) | 0.065 |
| Fusiform (L) | 11240.81 ± 811.23 (10079.27 - 12971.56) | 11391.31 ± 1608.90 (9255.54 - 14392.37) | 11328.61 ± 1048.78 (9884.41 - 13058.16) | 0.632 |
| Fusiform (R) | 12005.13 ± 1046.06 (10287.48 - 13850.20) | 12144.68 ± 1467.49 (9979.94 - 14084.47) | 11398.67 ± 625.05 (10699.07 - 12391.21) | 0.326 |
| Postcentral (L) | 10185.37 ± 1285.77 (8335.50 - 12477.85) | 10706.43 ± 1856.39 (8115.70 - 13595.44) | 10494.13 ± 1189.61 (9044.68 - 12480.62) | 0.604 |
| Postcentral (R) | 10367.49 ± 1478.83 (8488.16 - 13285.31) | 10557.03 ± 3286.17 (6253.72 - 15224.01) | 11824.37 ± 1331.88 (9412.87 -12799.91) | 0.398 |
| Parietal superior (L) | 6320.58 ± 815.94 (5241.50 - 8198.64) | 6045.19 ± 2062.18 (2959.52 - 9020.73) | 5550.44 ± 586.65 (4792.53 - 6585.64) | 0.358 |
| Parietal superior (R) | 5349.27 ± 1096.11 (3350.01 - 7753.99) | 6197.43 ± 1993.29 (4265.74 - 9218.41) | 4949.72 ± 766.19 (4111.24 - 5929.04) | 0.197 |
| Parietal inferior (L) | 10776.71 ± 1463.27 (8832.10 - 13164.08) | 11283.96 ± 1443.32 (9186.70 - 13620.23) | 10287.36 ± 1855.49 (7684.52 - 13035.26) | 0.158 |
| Parietal inferior (R) | 6091.84 ± 1230.44 (3434.46 - 8400.20) | 5797.69 ± 1317.01 (3321.84- 7424.01) | 5673.43 ± 881.67 (4403.53 - 6987.08) | 0.710 |
| Supramarginal (L) | 4667.80 ± 714.91 (3434.14 – 5576.18) | 4925.52 ± 964.12 (3948.34 – 6329.62) | 4584.44 ± 493.16 (3834.54 – 5146.63) | 0.568 |
| Supramarginal (R) | 7334.60 ± 1140.53 (5518.13 – 9157.04) | 6650.51 ± 2115.52 (4317.59 – 11125.72) | 7381.71 ± 681.51 (6540.59 – 8332.95) | 0.567 |
| Angular gyrus (L) | 5511.19 ± 974.94 (3783.56 – 7712.64) | 4495.95 ± 988.50 (3209.76 – 5934.27) | 5207.49 ± 1089.91 (3777.44 – 6582.00) | 0.098 |
| Angular gyrus (R) | 7040.42 ± 1065.16 (5265.16 – 9083.05) | 6652.16 ± 1075.61 (5319.75 – 8519.70) | 5754.00 ± 950.83 (4644.95 – 6901.46) * | **0.041** |
| Precuneus (L) | 14204.36 ± 1086.44 (11417.25 – 15686.32) | 13374.92 ± 1793.44 (10359.73 – 15760.25) | 12277.03 ± 1282.93 (11060.42 – 13922.88) ***** | **0.003** |
| Precuneus (R) | 11056.32 ± 1060.83 (8678.08 – 12993.69) | 10360.62 ± 1385.89 (8544.75 – 12783.08) | 10468.78 ± 982.07 (9161.95 – 11486.59) | 0.219 |
| Paracentral lobule (L) | 2919.91 ± 495.80 (2073.61 - 3743.57) | 3023.89 ± 504.62 (2602.64 - 4068.61) | 2926.93 ± 340.11 (2320.17 - 3273.66) | 0.073 |
| Paracentral lobule (R) | 1525.84 ± 360.18 (1001.52 - 2162.34) | 1730.12 ± 523.20 (1111.07 - 2774.35) | 1839.96 ± 317.18 (1323.00 - 2276.42) | 0.211 |
| Caudate (L) | 4937.36 ± 488.58 (4128.65 - 5609.72) | 5139.49 ± 366.20 (4615.27 - 5693.84) | 5390.95 ± 564.99 (4631.30 - 6177.59) | 0.189 |
| Caudate (R) | 5210.72 ± 482.43 (4524.21 - 6200.00) | 5406.43 ± 486.77 (4902.39 - 6259.12) | 5406.43 ± 486.77 (4902.39 - 6259.12) | 0.250 |
| Putamen (L) | 5131.97 ± 633.02 (4271.30 - 6677.66) | 4987.99 ± 454.31 (4297.53 - 5731.97) | 5113.87 ± 435.94 (4458.13 - 5578.76) | 0.793 |
| Putamen (R) | 5128.08 ± 744.22 (3952.20 - 6775.85) | 4956.58 ± 532.63 (3969.77 - 5848.27) | 5050.23 ± 356.69 (4458.98 - 5426.80) | 0.674 |
| Pallidum (L) | 1842.70 ± 183.31 (1423.60 - 2207.61) | 1851.58 ± 133.34 (1637.90 - 2015.79) | 1877.54 ± 135.50 (1708.48 - 2102.13) | 0.919 |
| Pallidum (R) | 1791.68 ± 158.62 (1559.61 - 2093.87) | 1742.94 ± 165.92 (1535.21 - 1912.83) | 1824.87 ± 114.76 (1696.11 - 1956.60) | 0.658 |
| Thalamus (L) | 6955.20 ± 784.68 (5571.23 - 8445.60) | 6697.92 ± 451.56 (6205.32 - 7585.11) | 7082.14 ± 507.09 (6169.94 - 7548.24) | 0.620 |
| Thalamus (R) | 6810.24 ± 705.52 (5513.47 - 7952.86) | 6547.94 ± 350.67 (6188.49 - 7146.61) | 6864.72 ± 373.71 (6346.87 - 7381.45) | 0.561 |
| Heschl (L) | 1030.21 ± 213.04 (732.32 - 1381.01) | 1064.80 ± 255.75 (822.05 - 1540.62) | 1096.16 ± 195.56 (793.03 - 1331.63) | 0.749 |
| Heschl (R) | 1141.97 ± 233.09 (743.02 - 1521.03) | 1151.14 ± 274.55 (835.94 - 1552.86) | 1213.68 ± 269.61 (889.07 - 1553.95) | 0.787 |
| Temporal superior (L) | 9950.37 ± 1266.85 (8037.59 - 12757.30) | 9926.60 ± 1936.55 (7661.97 - 13610.87) | 10291.26 ± 841.89 (9291.97 - 11185.93) | 0.825 |
| Temporal superior (R) | 13361.69 ± 1576.48 (9983.72 - 15643.75) | 13674.37 ± 2241.32 (10360.28 - 17174.11) | 13859.17 ± 640.33 (12912.03 - 14620.18) | 0.692 |
| Temporal pole superior (L) | 4976.68 ± 776.68 (3355.57 - 6925.77) | 5365.02 ± 392.68 (4847.95 - 6061.66) | 5339.14 ± 407.18 (4728.76 - 5717.05) | 0.184 |
| Temporal pole superior (R) | 5471.76 ± 776.6 (4076.80 - 6941.94) | 5757.67 ± 897.13 (4516.10 - 7294.51) | 5622.43 ± 522.77 (4934.14 - 6121.42) | 0.542 |
| Temporal middle (L) | 20761.70 ± 1881.69 (18428.63 - 25104.44) | 21733.01 ± 2484.96 (18342.48 - 26263.36) | 21009.31 ± 2139.94 (19428.62 - 24359.56) | 0.343 |
| Temporal middle (R) | 20511.84 ± 1750.42 (16835.62 - 24043.96) | 21866.65 ± 2735.51 (17001.44 - 26251.21) | 20137.18 ± 930.18 (18894.65 - 21361.68) | 0.068 |
| Temporal pole middle (L) | 3410.20 ± 603.55 (2207.34 - 4214.14) | 3455.52 ± 500.86 (2612.29 - 4145.74) | 3430.24 ± 905.96 (2211.64 - 4687.60) | 0.979 |
| Temporal pole middle (R) | 4475.41 ± 898.55 (3244.18 - 6262.79) | 4305.34 ± 677.53 (3391.01 - 5418.35) | 4108.01 ± 324.29 (3482.36 - 4373.67) | 0.569 |
| Temporal inferior (L) | 15020.48 ± 1465.77 (13681.80 - 18455.95) | 15039.12 ± 1842.64 (12421.29 - 17999.25) | 14218.46 ± 801.55 (13410.22 - 15300.48) | 0.374 |
| Temporal inferior (R) | 16186.66 ± 1429.17 (14173.37 - 19200.56) | 16773.80 ± 1845.90 (14668.32 - 19919.28) | 16661.90 ± 1964.17 (13458.86 - 13458.86) | 0.571 |

Volumes are expressed in mm^3^. Values are reported as means ± standard deviations [min. value – max. value]. P values refer to age-, sex- and MR scanner-adjusted ANOVA models, followed by post-hoc pairwise comparisons, Bonferroni-corrected for multiple comparisons. Abbreviations: GM= grey matter; L= left; R= right; sMND= sporadic motor neuron disease. Symbols: *= significantly different from sMND.

**Supplementary Table 3.** Diffusion tensor (DT) MRI metrics of WM tracts in MND patients and controls**.**

|  | **Healthy Controls** | **sMND** | ***TARDBP* MND** | **p** |
| --- | --- | --- | --- | --- |
| **CC** |  | | | |
| FA | 0.49 ± 0.18 (0.46 – 0.53) | 0.47 ± 0.03 (0.41 – 0.49) | 0.48 ± 0.01 (0.46 –0.49) | 0.107 |
| MD [x10^-3^ mm^2^ s^-1^] | 0.86 ± 0.08 (0.08 - 1.05) | 0.88 ± 0.05 (0.79 - 0.98) | 0.89 ± 0.08 (0.765- 1.09) | 0.395 |
| radD [x10^-3^ mm^2^ s^-1^] | 0.61 ± 0.06 (0.534 –0.77) | 0.64 ± 0.05 (0.56 - 0.76) | 0.64 ± 0.07 (0.541 – 0.787) | 0.234 |
| axD [x10^-3^ mm^2^ s^-1^] | 1.37 ± 0.09 (1.21 – 1.61) | 1.37 ± 0.06 (1.25 – 1.60) | 1.39 ± 0.12 (1.,21 – 1.70) | 0.686 |
| **CC precentral** |  |  |  |  |
| FA | 0.46 ± 0.04 (0.35 – 0.51) | 0.43 ± 0.03 (0.36 – 0.47) | 0.44 ± 0.02 (0.41 – 0.47) | 0.335 |
| MD [x10^-3^ mm^2^ s^-1^] | 0.86 ± 0.06 (0.75 – 1.01) | 0.91 ± 0.07 (0.81 – 1.03) | 0.88 ± 0.06 (0.80 – 0.98) | 0.310 |
| radD [x10^-3^ mm^2^ s^-1^] | 0.64 ± 0.07 (0.52 – 0.84) | 0.69 ± 0.07 (0.59 - 0.81) | 0.66 ± 0.06 (0.57 - 0.76) | 0.381 |
| axD [x10^-3^ mm^2^ s^-1^] | 1.310 ± 0.05 (1.21 - 1.40) | 1.35 ± 0.06 (1.23 - 1.47) | 1.31 ± 0.06 (1.23 - 1.41) | 0.275 |
| **CC premotor** |  |  |  |  |
| FA | 0.43 ± 0.02 (0.34 - 0.48) | 0.42 ± 0.02 (0.37 - 0.44) | 0.44 ± 0.03 (0.41 - 0.49) | 0.654 |
| MD [x10^-3^ mm^2^ s^-1^] | 0.86 ± 0.06 (0.78 - 1.02) | 0.86 ± 0.05 (0.77 - 0.93) | 0.82 ± 0.042 (0.76 - 0.88) | 0.175 |
| radD [x10^-3^ mm^2^ s^-1^] | 0.65 ± 0.07 (0.57 - 0.85) | 0.65 ± 0.05 (0.57 - 0.74) | 0.61 ± 0.04 (0.54 - 0.67) | 0.268 |
| axD [x10^-3^ mm^2^ s^-1^] | 1.30 ± 0.07 (1.20 - 1.47) | 1.28 ± 0.06 (1.18 - 1.36) | 1.26 ± 0.04 (1.19 - 1.33) | 0.143 |
| **CC supplementary motor** |  |  |  |  |
| FA | 0.50 ± 0.03 (0.38 - 0.53) | 0.48 ± 0.04 (0.39 - 0.51) | 0.48 ± 0.02 (0.44 - 0.52) | 0.460 |
| MD [x10^-3^ mm^2^ s^-1^] | 0.81 ± 0.05 (0.75 - 0.96) | 0.84 ± 0.05 (0.78 - 0.94) | 0.82 ± 0.03 (0.77 - 0.89) | 0.762 |
| radD [x10^-3^ mm^2^ s^-1^] | 1.30 ± 0.05 (1.22 - 1.45) | 1.32 ± 0.04 (1.25 - 1.38) | 1.30 ± 0.04 (1.22 - 1.38) | 0.689 |
| axD [x10^-3^ mm^2^ s^-1^] | 0.56 ± 0.06 (0.51 - 0.78) | 0.60 ± 0.06 (0.54 - 0.73) | 0.58 ± 0.04 (0.52 - 0.64) | 0.890 |
| **Cingulate (L)** |  |  |  |  |
| FA | 0.36 ± 0.02 (0.33 - 0.40) | 0.37 ± 0.02 (0.34 - 0.39) | 0.38 ± 0.02 (0.35 - 0.42) | 0.207 |
| MD [x10^-3^ mm^2^ s^-1^] | 0.86 ± 0.04 (0.79 - 0.97) | 0.87 ± 0.02 (0.84 - 0.90) | 0.85 ± 0.03 (0.78 - 0.90) | 0.615 |
| radD [x10^-3^ mm^2^ s^-1^] | 0.69 ± 0.05 (0.61 - 0.81) | 0.69 ± 0.02 (0.66 - 0.73) | 0.67 ± 0.04 (0.59 - 0.73) | 0.412 |
| axD [x10^-3^ mm^2^ s^-1^] | 1.20 ± 0.04 (1.15 -1.29) | 1.22 ± 0.03 (1.193 - 1.28) | 1.20 ± 0.03 (1.16 - 1.24) | 0.967 |
| **Cingulate (R)** |  |  |  |  |
| FA | 0.36 ± 0.01 (0.33 - 0.39) | 0.36 ± 0.01 (0.34 - 0.38) | 0.37 ± 0.02 (0.36 - 0.42) | 0.248 |
| MD [x10^-3^ mm^2^ s^-1^] | 0.84 ± 0.03 (0.80 - 0.90) | 0.86 ± 0.04 (0.79 - 0.93) | 0.83 ± 0.03 (0.78 - 0.87) | 0.495 |
| radD [x10^-3^ mm^2^ s^-1^] | 0.68 ± 0.03 (0.63 - 0.74) | 0.69 ± 0.04 (0.62 - 0.76) | 0.66 ± 0.03 (0.59 - 0.70) | 0.345 |
| axD [x10^-3^ mm^2^ s^-1^] | 1.17 ± 0.03 (1.12 - 1.22) | 1.20 ± 0.05 (1.13 - 1.28) | 1.18 ± 0.03 (1.14 - 1.22) | 0.782 |
| **Inferior longitudinal fasciculus (L)** |  |  |  |  |
| FA | 0.44 ± 0.02 (0.39 - 0.48) | 0.43 ± 0.02 (0.38 - 0.47) | 0.45 ± 0.05 (0.39 - 0.55) | 0.506 |
| MD [x10^-3^ mm^2^ s^-1^] | 0.79 ± 0.02 (0.74 - 0.83) | 0.83 ± 0.04 (0.77 - 0.91) | 0.83 ± 0.04 (0.75 - 0.88) | **0.035** |
| radD [x10^-3^ mm^2^ s^-1^] | 0.59 ± 0.03 (0.53 - 0.64) | 0.63 ± 0.04 (0.56 - 0.70) | 0.61 ± 0.06 (0.48 - 0.68) | 0.130 |
| axD [x10^-3^ mm^2^ s^-1^] | 1.21 ± 0.02 (1.16 - 1.25) | 1.24 ± 0.05 (1.16 - 1.36) | 1.27 ± 0.04 (1.20 - 1.32) * | **0.011** |
| **Inferior longitudinal fasciculus (R)** |  |  |  |  |
| FA | 0.43 ± 0.03 (0.38 - 0.48) | 0.44 ± 0.02 (0.40 - 0.47) | 0.44 ± 0.04 (0.39 - 0.49) | 0.864 |
| MD [x10^-3^ mm^2^ s^-1^] | 0.82 ± 0.03 (0.78 - 0.89) | 0.85 ± 0.03 (0.82 - 0.91) | 0.86 ± 0.03 (0.80 - 0.91) | 0.063 |
| radD [x10^-3^ mm^2^ s^-1^] | 0.61 ± 0.04 (0.56 - 0.71) | 0.63 ± 0.03 (0.60 - 0.70) | 0.65 ± 0.05 (0.58 - 0.72) | 0.254 |
| axD [x10^-3^ mm^2^ s^-1^] | 1.23 ± 0.04 (1.17 - 1.30) | 1.28 ± 0.04 (1.21 - 1.34) * | 1.29 ± 0.03 (1.25 - 1.33) * | **0.007** |
| **Superior longitudinal fasciculus (L)** |  |  |  |  |
| FA | 0.45 ± 0.01 (0.42 - 0.48) | 0.44 ± 0.02 (0.39 - 0.46) | 0.45 ± 0.02 (0.42 - 0.51) | 0.457 |
| MD [x10^-3^ mm^2^ s^-1^] | 0.75 ± 0.02 (0.71 - 0.81) | 0.77 ± 0.03 (0.72 - 0.84) | 0.77 ± 0.03 (0.73 - 0.83) | 0.219 |
| radD [x10^-3^ mm^2^ s^-1^] | 0.56 ± 0.02 (0.53 - 0.61) | 0.58 ± 0.58 (0.53 - 0.66) | 0.57 ± 0.03 (0.51 - 0.63) | 0.302 |
| axD [x10^-3^ mm^2^ s^-1^] | 1.14 ± 0.03 (1.09 - 1.21) | 1.16 ± 0.04 (1.11 - 1.21) | 1.170 ± 0.03 (1.13 - 1.24) | 0.205 |
| **Superior longitudinal fasciculus (R)** |  |  |  |  |
| FA | 0.43 ± 0.02 (0.38 - 0.46) | 0.43 ± 0.02 (0.39 - 0.47) | 0.44 ± 0.03 (0.37 - 0.48) | 0.926 |
| MD [x10^-3^ mm^2^ s^-1^] | 0.77 ± 0.03 (0.71 - 0.83) | 0.78 ± 0.03 (0.73 - 0.86) | 0.77 ± 0.03 (0.74 - 0.83) | 0.696 |
| radD [x10^-3^ mm^2^ s^-1^] | 0.58 ± 0.03 (0.53 - 0.66) | 0.59 ± 0.04 (0.53 - 0.68) | 0.57 ± 0.04 (0.53 - 0.66) | 0.794 |
| axD [x10^-3^ mm^2^ s^-1^] | 1.15 ± 0.03 (1.07 - 1.19) | 1.17 ± 0.04 (1.12 - 1.23) | 1.17 ± 0.02 (1.11 - 1.19) | 0.446 |
| **Uncinate fasciculus (L)** |  |  |  |  |
| FA | 0.38 ± 0.02 (0.34 - 0.42) | 0.36 ± 0.02 (0.31 - 0.39) | 0.38 ± 0.02 (0.34 - 0.40) | 0.373 |
| MD [x10^-3^ mm^2^ s^-1^] | 0.84 ± 0.05 (0.75 - 0.95) | 0.88 ± 0.07 (0.80 - 1.03) | 0.86 ± 0.03 (0.82 - 0.93) | 0.385 |
| radD [x10^-3^ mm^2^ s^-1^] | 0.66 ± 0.05 (0.56 - 0.78) | 0.71 ± 0.08 (0.62 - 0.87) | 0.67 ± 0.04 (0.63 - 0.75) | 0.344 |
| axD [x10^-3^ mm^2^ s^-1^] | 1.21 ± 0.05 (1.12 - 1.29) | 1.24 ± 0.06 (1.15 - 1.37) | 1.24 ± 0.03 (1.20 - 1.28) | 0.391 |
| **Uncinate fasciculus (R)** |  |  |  |  |
| FA | 0.39 ± 0.02 (0.34 - 0.42) | 0.40 ± 0.03 (0.34 - 0.44) | 0.39 ± 0.02 (0.36 - 0.42) | 0.980 |
| MD [x10^-3^ mm^2^ s^-1^] | 0.84 ± 0.04 (0.75 - 0.94) | 0.83 ± 0.05 (0.77 - 0.96) | 0.85 ± 0.03 (0.81 - 0.90) | 0.802 |
| radD [x10^-3^ mm^2^ s^-1^] | 0.651 ± 0.04 (0.57 - 0.74) | 0.64 ± 0.06 (0.59 - 0.78) | 0.66 ± 0.03 (0.61 - 0.71) | 0.924 |
| axD [x10^-3^ mm^2^ s^-1^] | 1.22 ± 0.04 (1.13 - 1.32) | 1.25 ± 0.04 (1.20 - 1.33) | 1.25 ± 0.04 (1.20 - 1.33) | 0.558 |
| **CST-L** |  | | | |
| FA | 0.53 ± 0.02 (0.50 – 0.58) | 0.52 ± 0.03 (0.47 – 0.56) | 0.51 ± 0.05 (0.43 – 0.59) | 0.236 |
| MD [x10^-3^ mm^2^ s^-1^] | 0.77 ± 0.05 (0.68 - 0.85) | 0.80 ± 0.03 (0.75 - 0.89) | 0.79 ± 0.04 (0.73 -0.89) | 0.236 |
| radD [x10^-3^ mm^2^ s^-1^] | 0.52 ± 0.04 (0.42 - 0.58) | 0.55 ± 0.03 (0.51 - 0.59) | 0.54 ± 0.03 (0.51 - 0.61) | 0.181 |
| axD [x10^-3^ mm^2^ s^-1^] | 1.26 ± 0.08 (1.14 – 1.41) | 1.20 ± 0.07 (1.23 – 1.49) | 1.27 ± 0.09 (1.18 – 1.48) | 0.371 |
| **CST - R** |  | | | |
| FA | 0.55 ± 0.03 (0.49 – 0.60) | 0.52 ± 0.04 (0.42 – 0.57) | 0.51 ± 0.03 (0.45 – 0.60) ***** | **0.025** |
| MD [x10^-3^ mm^2^ s^-1^] | 0.77 ± 0.04 (0.72 - 0.91) | 0.83 ± 0.01 (0.74 -1.07) | 0,80 ± 0.03 (0.73 - 0.82) | 0.075 |
| radD [x10^-3^ mm^2^ s^-1^] | 0.52 ± 0.05 (0.46 - 0.66) | 0.58 ± 0.01 (0.50 - 0.84) | 0.54 ± 0.04 (0.47 - 0.59) | 0.061 |
| axD [x10^-3^ mm^2^ s^-1^] | 1.29 ± 0.07 (1.19 – 1.43) | 1.32 ± 0.01 (1.19 – 1.53) | 1.26 ± 0.06 (1.17 – 1.35) | 0.120 |

Values are reported as means ± standard deviations [min. value – max. value]. P values refer to age-, sex- and MR scanner-adjusted ANOVA models, followed by post-hoc pairwise comparisons, Bonferroni-corrected for multiple comparisons. Abbreviations: axD: axial diffusivity; CC= corpus callosum; CST= corticospinal tract; FA= fractional anisotropy; L=left; MD= mean diffusivity; R= right; radD= radial diffusivity; sMND= sporadic motor neuron disease. Symbols: *=significantly different from healthy controls.
